# In Silico Comparative Analysis of the Functional, Structural, and Evolutionary Properties of SARS-CoV-2 Variant Spike Proteins

**DOI:** 10.2196/37391

**Published:** 2022-05-30

**Authors:** Renukaradhya K Math, Nayana Mudennavar, Palaksha Kanive Javaregowda, Ambuja Savanur

**Affiliations:** 1 SDM Research Institute for Biomedical Sciences Shri Dharmasthala Manjunatheshwara University Dharwad India

**Keywords:** spike protein variants, NCBI, bioinformatics tools, pI, isoelectric point, 2D map, phylogenetic tree, COVID-19, COVID therapy, SARS-CoV-2 treatment, therapeutic, spike protein, protein, prophylactic, sequence analysis, genomic, bioinformatics, viral protein

## Abstract

**Background:**

A recent global outbreak of COVID-19 caused by the severe acute respiratory syndrome coronavirus-2 (SARS-CoV-2) created a pandemic and emerged as a potential threat to humanity. The analysis of virus genetic composition has revealed that the spike protein, one of the major structural proteins, facilitates the entry of the virus to host cells.

**Objective:**

The spike protein has become the main target for prophylactics and therapeutics studies. Here, we compared the spike proteins of SARS-CoV-2 variants using bioinformatics tools.

**Methods:**

The spike protein sequences of wild-type SARS-CoV-2 and its 6 variants—D614G, alpha (B.1.1.7), beta (B.1.351), delta (B.1.617.2), gamma (P.1), and omicron (B.1.1.529)—were retrieved from the NCBI database. The ClustalX program was used to sequence multiple alignment and perform mutational analysis. Several online bioinformatics tools were used to predict the physiological, immunological, and structural features of the spike proteins of SARS-CoV-2 variants. A phylogenetic tree was constructed using CLC software. Statistical analysis of the data was done using jamovi 2 software.

**Results:**

Multiple sequence analysis revealed that the P681R mutation in the delta variant, which changed an amino acid from histidine (H) to arginine (R), made the protein more alkaline due to arginine’s high pKa value (12.5) compared to histidine’s (6.0). Physicochemical properties revealed the relatively higher isoelectric point (7.34) and aliphatic index (84.65) of the delta variant compared to other variants. Statistical analysis of the isoelectric point, antigenicity, and immunogenicity of all the variants revealed significant correlation, with *P* values ranging from <.007 to .04. The generation of a 2D gel map showed the separation of the delta spike protein from a grouping of the other variants. The phylogenetic tree of the spike proteins showed that the delta variant was close to and a mix of the *Rousettus* bat coronavirus and MERS-CoV.

**Conclusions:**

The comparative analysis of SARS-CoV-2 variants revealed that the delta variant is more aliphatic in nature, which provides more stability to it and subsequently influences virus behavior.

## Introduction

The constant evolution of new variants of SARS-CoV-2 is a substantial challenge to people from every sector, particularly those in health care and research and development in the areas of diagnostics, prophylactics, and therapeutics development, as well as policy makers and administrators. The virus was first observed in December 2019 in China and later spread throughout the globe causing a pandemic. However, continuous mutations in the genome of the virus have created several new variants among the circulating viruses in different geographical regions worldwide. These mutations have led to increased transmissibility, antibody evasion, and severity in patients. Several research studies have cited that the spike protein of the coronavirus is responsible for interacting with human cells to penetrate inside [1,2]; however, adaptive mutations accumulated in the gene responsible for the spike protein have allowed the virus to acclimatize and escape the host immune system. A number of variants have been isolated and identified worldwide; the World Health Organization classified them as variants of concern (alpha, beta, gamma, delta, and omicron) and variants of interest (VOIs; eta, iota, kappa, and lambda) [3].

A single mutation in a gene can lead to a change of an amino acid in a protein, which can drastically affect an organism’s ability in many ways, such as transmissibility, quick adaptability, stability evading the host immune system, and pathogenicity [4]. The availability of high throughput second generation sequencing technologies like next-generation sequencing in the last decade has helped in the timely sequencing of the genomes of SARS-CoV-2 variants to identify mutations occurring among new genetic variants. Rapid sequencing technology has not only facilitated sequencing of the viral genome but also helped accelerate the development of diagnostic tools, vaccines, and therapeutics for COVID-19. Furthermore, the availability of genome sequence data and databases helped the scientific community understand the virus through the molecular, biochemical, genome, and proteome analyses of the virus. However, the continuous evolution of SARS-CoV-2 compels the scientific community to continuously strive toward new and thorough understanding of the properties of the virus variants and develop counter strategies against the virus to safeguard humankind.

The isoelectric point (pI) of a protein is crucial in determining the physicochemical properties of the protein [5]. The exposure of charged amino acids on the protein surface to solvents, hydration, and dehydration also influences the pI of a protein [6,7]. Thus, evaluation of the pI of the spike proteins of wild-type and mutated variants is quintessential in understanding the influence of the spike protein on virus behavior and the transmission rate of viruses, as well as developing prophylactic and therapeutic agents. Additionally, the roles of posttranslational modification, phosphorylation, methylation, and alkylation also influence the pI of a protein, which cannot be ignored. Similarly, the genome of SARS-CoV-2 is prone to genetic evolution or genetic shift while adapting to a human host’s microenvironment. Such mutations result in the emergence of new variants that might have different characteristics compared to its ancestral strains. Therefore, in this study, we aim to adopt an in silico method to analyze the spike protein sequences of wild-type and other variants of SARS-CoV-2 to know their physicochemical, functional, and evolutionary properties, which might help in the surveillance of SARS-CoV-2 through a systematic understanding of the continuously evolving properties as well as to develop the diagnostic tools and standardization of prophylactics and therapeutics strategies.

## Methods

### Retrieval of Spike Protein Sequences and Multiple Sequence Alignment

The protein sequences of wild-type SARS-CoV-2 and its 6 variants—D614G, alpha (B.1.1.7), beta (B.1.351), delta (B.1.617.2), gamma (P.1), and omicron (B.1.1.529)—were obtained from the NCBI database. The list of all the variants with the NCBI accession numbers are mentioned in Table 1. Multiple sequence alignment of all the protein was done using ClustalX2 software [8] to identify and confirm common and specific mutations among all the sequences listed in Table 1.

**Table 1:** List of SARS-CoV-2 spike proteins and the comparison of mutational analysis data of the wild type and its variants.

SARS-CoV-2 variants	First identified	NCBI accession	Linear SeqVrl	Pango lineage	Mutation in spike protein variants sequence	Specific mutation in spike protein variants
Wild type	Wuhan, China	YP_009724390	—^a^	—	—	—
D614G	United States	QTA38988	21-Mar-21	D614G	D614G	D614G
Alpha (B.1.1.7)	United Kingdom	P0DTC2	02-Jun-21	B.1.1.7	E484K, A570D, D614G, P681H	N501Y
Beta (B.1.351)	South Africa	7LYQ_C	09-Jul-21	B.1.351	K417N, E484K, N501Y, D614G	R246I
Delta (B.1.617.2)	India	QWP92316	12-Jun-21	B.1.617.2	E156, K417N, D614G, N501Y	T19R, L452R, T478K, P581R, P681R, D950N
Gamma (P.1)	Brazil	7M8K_C	28-May-21	P.1	K417N, E484K, N501Y, D614G	T20N, P26S, D138Y, R190S, T1027I
Omicron (B.1.1.529)	South Africa	7QO7_C	19-Jan-2022	B.1.1.529	N501Y, Y505H, T547K, D614G, H655Y, etc	A67V, del69-70, T95I, del142-144, Y145D, etc

^a^Not available.

### Physicochemical Properties and Posttranslational Modification Prediction

Physicochemical properties such as total amino acids, molecular weight, pI, grand average of hydropathicity (GRAVY), aliphatic index, etc, were predicted using the ProtParam tool [9]. Posttranslational modifications for all spike protein variants were predicted as follows: phosphorylation sites using the NetPhos 2.0 server [10]; glycosylation sites using the NetNGlyc 1.0 server [11]; and disulfide bonds using the Scratch Protein Predictor server [12].

### Prediction of Immunoproperties

B-cell epitopes of all the variants were predicated using the ABCpred server [13] and exposed B-cell epitopes were predicted using the BepiPred 2.0 server [14]. T-cell epitopes and their immunogenicity were predicted using the IEBD Analysis Resource server [15], strong binding T-cells were identified using the NetMHCpan 4.1 server [16], and predications of cytotoxic T-lymphocytes were identified by the NetCTL 4.0 server [17].

### Secondary and Tertiary Structure Prediction

Predications of secondary structures were identified by the PHYRE2 server [18] and the percentage of the following parameters were assessed: alpha helix, beta stand, transmembrane helix, and disorder. Subsequently, the tertiary structure was predicted using the Swiss model server [19] and the global model quality estimation, confidence, and coverage scores were collected. The structure was also analyzed for Ramachandran plot–allowed regions.

### Generation of Phylogenetic Tree and 2D Gel Reference Map

The spike protein sequences of the wild type and variants were collected from NCBI, and the protein sequences of MERS-CoV and *Rousettus* bat coronavirus GCCDC1 were also obtained from NCBI with accession numbers AHY22525 and QKF94914, respectively. The phylogenetic tree was constructed using CLC sequence viewer and DNAMAN software [20] by neighbor joining method, and a virtual 2D protein map of all the spike protein variants was generated using the JVirGel V2.0 software [21].

### Ethical Considerations

The study is registered with the institutional ethics committee of the Shri Dharmasthala Manjunatheshwara University (registration no. ECR/950/Inst/KA/2017/RR-21), Sattur, India. According to the institutional ethics committee guidelines, in compliance with the National guidelines/regulation on ethics in Biomedical Research, ethical clearance is not required for studies not involving human subjects/animals/patient medical records/tissues/biologicals fluids/pathogenic microorganism.

## Results

### In Silico Analysis

The results of an in silico analysis of the wild-type and variant protein sequences of spike protein revealed several common and specific mutations as listed in Table 1. Interestingly, the mutation P681H in the alpha variant [22] changed an amino acid from histidine (H) to arginine (R). Further, the analysis of physicochemical properties revealed that the pI and antigenicity of the delta variant were relatively high compared to other variants, and immunoproperties like B- and T-cell epitope sequences were different in the beta and delta variant compared to others. The phylogenetic tree and 2D gel maps clearly separated the delta variant from others.

### Physicochemical Properties and Posttranslational Modification Prediction

The analysis of the physicochemical properties of all the spike proteins was interesting, especially the pI and aliphatic index, which ranged from 6.28 to 7.34 and from 82.04 to 84.65, respectively (Table 2). Among all the proteins, the delta variant had high pI (7.34) and high aliphatic index (84.65) when compared to other proteins (Table 2). However, all the proteins were stable. Meanwhile, the predicted total number of phosphorylation sites in the wild type was 133 whereas this prediction relatively decreased in the delta variant protein (Table 3). In addition, the number of serine, threonine, and tyrosine in phosphorylation sites varied among the variants compared to the wild type.

Predictions of the total number of N-glycosylation and disulfide bonds among the spike protein variants revealed numbers ranging from 16 to 20 and from 14 to 15 sites, respectively (Table 3).

**Table 2:** Comparison of predicted physicochemical property values of the spike protein of SARS-CoV-2 and its variants.

SARS-CoV-2 variants	Total amino acids	MW^a^	pI^b^	Extinction coefficient (M^-1^ cm^-1^)	EC/A^c^	Half-life (h)	Instability index	Classification of protein	Aliphatic index	GRAVY^d^
Wild type	1273	141178	6.24	148960	1.055	30	33.01	stable	84.67	–0.079
D614G	1252	138712	6.49	147345	1.062	30	32.06	stable	85.01	–0.067
Alpha (B.1.1.7)	1273	141178	6.24	148960	1.055	30	33.01	stable	84.67	–0.079
Beta (B.1.351)	1288	142201	6.38	148335	1.043	30	31.29	stable	82.03	–0.142
Delta (B.1.617.2)	1271	140895	7.34	148960	1.057	30	32.58	stable	84.65	–0.08
Gamma (P.1)	1257	139207	6.18	140315	1.008	30	31.15	stable	83.43	–0.127
Omicron (B.1.1.529)	1285	142424	6.63	146845	1.018	30	33.10	stable	81.84	–0.164

^a^MW: molecular weight.

^b^pI: isoelectric point.

^c^EC/A: extinction coefficient/absorbance for 1% solutions.

^d^GRAVY: grand average of hydropathicity.

**Table 3:** Comparison of phosphorylation, N-glycosylation, and disulfide bonds values of the spike protein of SARS CoV-2 and its variants.

SARS-CoV-2 variants	Phosphorylation				N-glycosylation			Disulfide bonds	
	Phosphorylation sites	Predicted Ser^a^, Thr^b^, and Tyr^c^ sites	Predicted number	N-glycosylation sites		Predicted number	Total number of cysteine		Predicted number
Wild type	Ser, Thr, Tyr	Ser-64, Thr-44, Tyr-22	133	Asn^d^-Ser/Thr		17	40		15
D614G	Ser, Thr, Tyr	Ser-64, Thr-45, Tyr-22	131	Asn-Ser/Thr		17	38		15
Alpha (B.1.1.7)	Ser, Thr, Tyr	Ser-67, Thr-44, Yyr-22,	133	Asn-Ser/Thr		17	40		15
Beta (B.1.351)	Ser, Thr, Tyr	Ser-71, Thr-43, Tyr-24	136	Asn-Ser/Thr		17	30		14
Delta (B.1.617.2)	Ser, Thr, Tyr	Ser-65, Thr-43, Tyr-22	130	Asn-Ser/Thr		16	40		15
Gamma (P.1)	Ser, Thr, Tyr	Ser-66, Thr-43, Tyr-24	133	Asn-Ser/Thr		20	30		14
Omicron (B.1.1.529)	Ser, Thr, Tyr	Ser-68, Thr-43, Tyr-21	132	Asn-Ser/Thr		18	30		14

^a^Ser: serine.

^b^Thr: threonine.

^c^Tyr: tyrosine.

^d^Asn: asparagine.

### Prediction of Immunoproperties

Predictions of the number of exposed B-cell epitopes for spike protein varied from 38 to 41 (Table 4). The B-cell epitope sequence for the wild-type, alpha, and gamma variants was QTQTNSPRRARSV, whereas this sequence changed for the D614G, beta, and delta variants. Among all the variants, the delta variant showed the highest score for protective antigen (0.4709) and antigenicity (0.7440; Table 4). Predictions for C-cell epitopes revealed that the number of epitopes ranged from 35 to 38 and the number of strong binders in T-cell were from 21 to 23. The predicted T-cell epitopes for the wild-type and gamma spike variants were identical to IGINITRFQTLLALH but changed in other spike protein variants. However, except in the alpha variant, the sequence QTLLALH was supposed to be conserved (Table 4). The immunogenicity prediction scores for the spike protein variants varied. Meanwhile, statistical analysis of pI, antigenicity, and immunogenicity scores revealed the significance of the data, especially between alpha and wild type (*P*=.007), delta and wild type (*P*=.02), and delta and alpha (*P*=.02). The correlation matrix was positive with *P* values ranging from <.007 to .04 (Table 5).

**Table 4:** Comparison of the immunological properties of the spike protein of SARS-CoV-2 and its variants.

SARS-CoV-2 variants	Exposed B-cell epitopes	B-cell epitopes	Protective antigen prediction score	Predicted probability of antigenicity score	Number of epitopes identified in CTL^a^	Number of strong binders in T-cell	Predicted epitopes in T-cell	Immunogenicity predication score
Wild type	40	QTQTNSPRRARSV	0.4646	0.717053	37	21	IGINITRFQ**TLLA**LH	0.3751
D614G	40	YHKNNKS	0.4583	0.741478	35	22	ITRFQ**TLLA**	0.96257
Alpha (B.1.1.7)	40	QTQTNSPRRARSV	0.4646	0.717053	37	21	NGTHWFVTQRNFYEP	0.3019
Beta (B.1.351)	40	HPQFEKGGGSGGGGSG	0.4542	0.643558	38	23	QPYRVVVLSFE**LL**HA	1.23216
Delta (B.1.617.2)	38	SLGAENSVAYSN	0.4709	0.744007	35	22	IRAAEIRASAN**LAA**T	0.0304
Gamma (P.1)	41	QTQTNSPRRARSV	0.4583	0.596261	36	22	IGINITRFQ**TLLAL**H	1.07515
Omicron (B.1.1.529)	33	QTQTKSHGSASSVA	0.4646	0.717053	31	20	IGINITRFQTLLALH	0.49637

^a^CTL: cytotoxic T-lymphocyte.

**Table 5:** The *P* values of isoelectric point, antigenicity, and immunogenicity of the spike protein of SARS-CoV-2 and its variants.

SARS-CoV-2 variants		Wild type	D614G	Alpha	Beta	Delta	Gamma	Omicron
**Wild type**								
	*r*	—^a^	0.996	1.000	0.989	0.999	0.992	1.000
	*P* value	—	.06	.007	.09	.02	.08	.01
**D614G**								
	*r*	0.996	—	0.995	0.998	0.992	0.999	0.998
	*P* value	.06	—	.06	.04	.08	.03	.04
**Alpha**								
	*r*	1.000	0.995	—	0.988	1.000	0.990	1.000
	*P* value	.007	.06	—	.10	.02	.09	.02
**Beta**								
	*r*	0.989	0.998	0.988	—	0.983	1.000	0.992
	*P* value	.09	.04	.10	—	.12	.01	.08
**Delta**								
	*r*	0.999	0.992	1.000	0.983	—	0.986	0.998
	*P* value	.02	.08	.02	.12	—	.11	.04
**Gamma**								
	*r*	0.992	0.999	0.990	1.000	0.986	—	0.994
	*P* value	.08	.03	.09	.01	.11	—	.07
**Omicron**								
	*r*	1.000	0.998	1.000	0.992	0.998	0.994	—
	*P* value	.01	.04	.02	.08	.04	.07	—

^a^Not applicable.

### Secondary and Tertiary Structure Prediction

**Figure 1:**
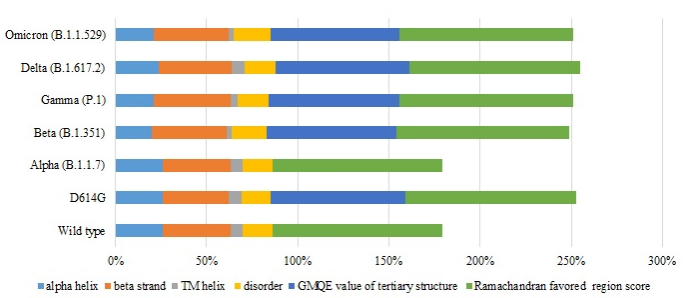
Graphical illustration of the predicted percentages of the secondary and tertiary structure and disorder of the spike proteins of SARS-CoV-2 wild type and its variants. GMQE: global model quality estimate; TM: transmembrane.

Secondary and tertiary prediction of all the spike protein variants showed that all protein structures were stable (Figure 1). The predicted proportion of alpha helices, beta strands, transmembrane helices, and disorders of all the variants are shown in Figure 1. The alpha helix percentages varied from 26% in the wild type to 21% in the gamma variant, whereas the beta strand percentages varied from 37% in the wild type to 42% in the gamma variant; however, analysis of alpha and beta percentages revealed that the proteins were stable. The global model quality estimate scores and Ramachandran favored regions—of which all the spike protein variants’ percentages were similar to the wild type’s—indicated that there was no significant change in the stability of the variants compared to the wild type (Figure 1).

### Generation of 2D Gel Reference Map and Phylogenetic Tree

**Figure 2:**
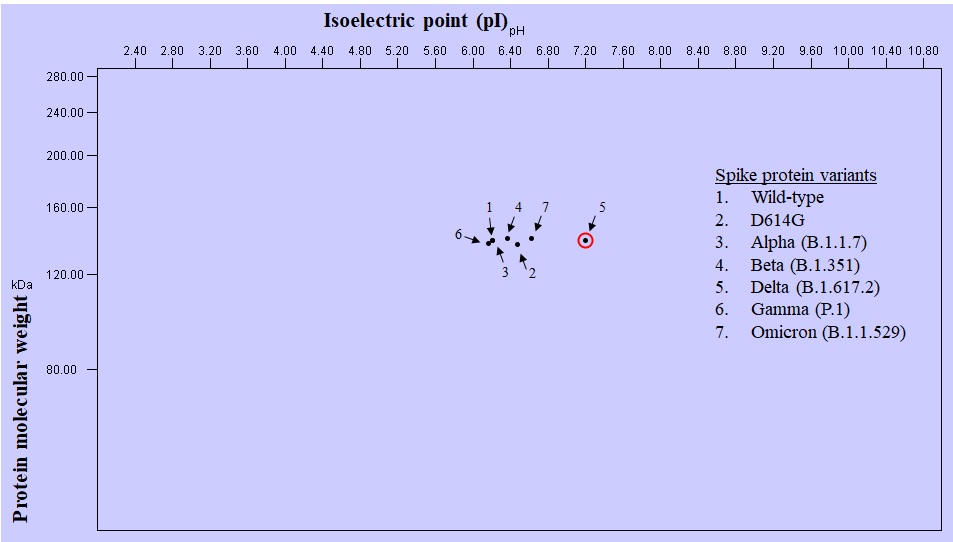
The 2D gel map of SARS-CoV-2 and its variants.

**Figure 3:**
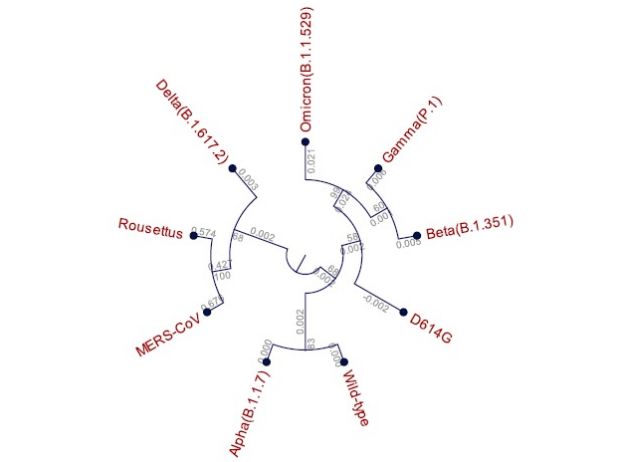
The phylogenetic tree of SARS-CoV-2 and its variants represented in circular cladogram. Protein sequences of MERS-CoV and *Rousettus* bat coronavirus were used as nearest relatives.

The 2D reference map of the spike protein and its variants revealed a grouping of the wild-type, D614G, alpha, beta, gamma, and omicron variants whereas the delta variant was clearly separated (Figure 2). The phylogenetic tree of the spike protein of the wild-type and variant proteins along with MERS-CoV and bat coronavirus was constructed as seen in Figure 3. Construction of the phylogenetic tree grouped the wild-type and alpha variants as one cluster and the beta, gamma, and omicron variants as another cluster. The D614G variant stood in between these 2 groups. Interestingly, the delta variant was closely grouped with MERS-CoV and *Rousettus* bat coronavirus. Meanwhile, the omicron variant showed 99% bootstrap values with the D614G variant, showing the closest relationship among all others. However, the tree has 2 branches: one with the wild type and variants (except delta) and another with the delta variant along with MERS-CoV and *Rousettus* bat coronavirus (Figure 3).

## Discussion

The current COVID-19 pandemic caused by SARS-CoV-2 has clearly demonstrated the potential of the coronavirus to continuously evolve in the wild and transmit to new species including humans with varied physicochemical and virulence properties. The world has witnessed several mutants of this virus, with “VOIs” and “variants of concern” taking several millions lives. However, the situation seems to be under control because of the availability of vaccines recently from the beginning of the year 2021. However, it is quintessential to understand the constantly mutating property of viruses to establish effective and timely health care strategies to avoid the consequential economic loss or burden and safeguard humankind. Understanding life-threatening organism such as SARS-CoV-2 is a continuous and challenging process for the health care and scientific community as well as for policy makers and administrators. There are several methods to study and understand the disease-causing agents, of which the in silico analysis method has contributed enormously to make the scientific community’s tasks easier by reducing the required effort, time, and costs in recent years. Therefore, in this study, we have made an effort to understand the variants of concerns, especially how their spike proteins spread around the globe. The analysis of the physicochemical properties of spike proteins of the wild-type and mutated variants of SARS-CoV-2 revealed interesting features of the delta variant, such as pI, molecular weight, instability index, GRAVY, and aliphatic index (Table 2).

The pI of a protein is crucial to understanding its biochemical function [23] and dependent on the dissociation and constant of the ionizable amino acid groups. The major ionizable amino acid groups present in SARS-CoV-2 are arginine, aspartate, histidine, glutamate, cysteine, and lysine, which play an important role in defining the pI of a spike protein [24-26]. However, posttranslational modification influences the pI of a protein. The mutation of P681R in the delta variant [22] changed an amino acid from histidine to arginine, which sparked our curiosity to try to understand the potential impact of the change in amino acid on the properties of the spike protein in the alpha and delta variants. Interestingly, the pKa values of both amino acids greatly differed—histidine has a value of 6.0 (low basic) and arginine has a value 12.5 (high basic). Other specific mutations observed in the delta variant were also basic or aliphatic in nature (Table 1). Thus, if this property is substantial, then that would make the protein more basic. The mutation P681H in the alpha variant did not cause substantial changes in the behavior of virus, but the mutation P681R in the delta variant did cause substantial changes in the behavior of the virus in terms of transmissibility, pathogenicity, immune evasion, and the severity of the infection [22]. Conversely, a neutral mutation was observed in the D614G variant spike protein [27,28], where an acidic amino acid (D) was replaced with neutral one (G).

To see the effect of the pI on the biochemical properties, the wild-type and variant spike proteins were evaluated by predicting the 2D gel reference map, which interestingly demonstrated grouping except the separation of the delta protein on the map (Figure 2). The results of the physicochemical properties and 2D gel analysis revealed that the pI of a protein plays an important role in the behavior of proteins, especially where high pI turns protein more positive or aliphatic. Additionally, it is interesting to know that this property of protein is used in antibody preparation—to make a protein or its subunit more immunogenic [29,30]. Hence, we suppose that this property of proteins should be taken into consideration when designing and preparing prophylactic and therapeutic agents. Similarly, a recent study on the VOIs of SARS-CoV-2 virus particles that measured pI using chemical force microscope revealed VOIs have lower surface charge and hydrophobicity than the wild type, which might have played a role in VOIs having increased transmission ability [29]. Therefore, the pI of protein and the surface charge and hydrophobicity of viral particles are important factors to consider when designing prophylactics and therapeutics for any viral diseases [4,28,30,31].

Substantial changes in the physicochemical properties of the spike protein of wild-type SARS-CoV-2 and its variants has not been observed; however, a high number of phosphorylation sites (136) was observed in the beta variant and a low number (130) was observed in the delta variant. Meanwhile, a high number of N-glycosylation sites (20) was observed in the gamma variant and a low number (16) was observed in the delta variant. The total number of disulfide bonds among the wild type and variants did not vary much; however, it ranged from 14 to 15 and the number bonds were enough to give structural stability. Overall, the physicochemical properties did not differ markedly; however, the differences found were enough to influence the change in protein behavior and, subsequently, the behavior of the virus.

The antigenicity and immunogenicity scores of wild-type SARS-CoV-2 and its variants were interesting, especially the delta variant which showed high and low scores, respectively. Comparison of pI, antigenicity, and immunogenicity was made to evaluate the significance of the data; the *P* values were .007 between the alpha variant and wild type, .02 between the delta variant and wild type, and .02 between the delta and alpha variants. Overall, these *P* values suggest that the predicted values and the antigenic and immunogenic sequences are reliable. The number of exposed B-cell and cytotoxic T-lymphocyte epitopes were low in the delta variant compared to the wild-type and other variants of SARS-CoV-2. The exposed B-cell epitope sequence for the delta variant was SLGAENSVAYSN, and the change in epitope sequence showed that mutations did have an effect on the morphology of the virus.

The evolutionary relationship among the wild type and its variants was evaluated by constructing a phylogenetic tree using protein sequences. Interestingly, we found a clear grouping of the D614G, beta, and gamma variants, with bootstrap values of 100% for beta and gamma and 93% between D614G and beta/gamma. The delta variant was close to *Rousettus* bat coronavirus and MERS-CoV (Figure 3) with a bootstrap value of 68%. The alpha variant was close to the wild type with an 83% bootstrap value. Interestingly, we could observe a hybrid position of the delta variant between the wild type and the MERS-CoV and *Rousettus* bat coronavirus. Therefore, we suppose that the delta variant might be carrying characteristics from the wild type and other wild bat coronaviruses (Figure 3). In another interesting observation, the omicron variant seems to have evolved from the D614G variant with a bootstrap value 99%, as well as having imported some characteristics from the beta and gamma variants since omicron was also seen branching from these 2 variants with a 60% bootstrap value (Figure 3).

In conclusion, our study highlights that the accumulation of adaptive mutations in SARS-CoV-2 influenced the change in pI of the spike protein and, subsequently, the behavior of the virus. The prediction and comparison of the physicochemical properties of spike proteins of the wild type and its variants revealed that the delta variant displayed unique changes compared to the wild type. Evolutionary features showed a clear separation of the wild-type, alpha, and delta spike proteins and the grouping of the D614G, beta, and gamma spike proteins. Nevertheless, the continuous evolution of new SARS-CoV-2 strains demands further systematic understanding of its variants, which would not only help in developing the improvised rapid “antigen test” market but also in developing vaccines and therapeutics. Thus, more similar studies would unravel the important biochemical properties, evolutionary history, immunological behavior, and physiological properties of viruses.

## Abbreviations

GRAVY: grand average of hydropathicity
pI: isoelectric point
VOI: variant of interest
